# Metagenomics characterization of respiratory viral RNA pathogens in children under five years with severe acute respiratory infection in the Free State, South Africa

**DOI:** 10.1002/jmv.28753

**Published:** 2023-04-27

**Authors:** Ayodeji E. Ogunbayo, Milton T. Mogotsi, Hlengiwe Sondlane, Saheed Sabiu, Martin M. Nyaga

**Affiliations:** ^1^ Next Generation Sequencing Unit and Division of Virology Faculty of Health Sciences, University of the Free State Bloemfontein South Africa; ^2^ Department of Biotechnology and Food Science Durban University of Technology Durban South Africa

**Keywords:** children, metagenomics, nasopharyngeal swab, next‐generation sequencing, severe acute respiratory infection, viral RNA pathogens

## Abstract

Prompt detection of viral respiratory pathogens is crucial in managing respiratory infection including severe acute respiratory infection (SARI). Metagenomics next‐generation sequencing (mNGS) and bioinformatics analyses remain reliable strategies for diagnostic and surveillance purposes. This study evaluated the diagnostic utility of mNGS using multiple analysis tools compared with multiplex real‐time PCR for the detection of viral respiratory pathogens in children under 5 years with SARI. Nasopharyngeal swabs collected in viral transport media from 84 children admitted with SARI as per the World Health Organization definition between December 2020 and August 2021 in the Free State Province, South Africa, were used in this study. The obtained specimens were subjected to mNGS using the Illumina MiSeq system, and bioinformatics analysis was performed using three web‐based analysis tools; Genome Detective, One Codex and Twist Respiratory Viral Research Panel. With average reads of 211323, mNGS detected viral pathogens in 82 (97.6%) of the 84 patients. Viral aetiologies were established in nine previously undetected/missed cases with an additional bacterial aetiology (*Neisseria meningitidis)* detected in one patient. Furthermore, mNGS enabled the much needed viral genotypic and subtype differentiation and provided significant information on bacterial co‐infection despite enrichment for RNA viruses. Sequences of nonhuman viruses, bacteriophages, and endogenous retrovirus K113 (constituting the respiratory virome) were also uncovered. Notably, mNGS had lower detectability rate for severe acute respiratory syndrome coronavirus 2 (missing 18/32 cases). This study suggests that mNGS, combined with multiple/improved bioinformatics tools, is practically feasible for increased viral and bacterial pathogen detection in SARI, especially in cases where no aetiological agent could be identified by available traditional methods.

## 1 INTRODUCTION

Severe acute respiratory infections (SARI) are a leading cause of morbidity and mortality, especially in vulnerable new‐borns and young children.^1^, ^2^, ^3^ The detection of viruses and bacteria implicated in respiratory infections could be challenging due to their nonspecific clinical presentation nature and the possibility of co‐infection.^4^, ^5^ The traditional testing methods, such as culture, have limitations, including low sensitivity and are time demanding.^6^, ^7^ Also, molecular approaches such as real‐time multiplex PCR (mRT‐PCR) can only detect targeted pathogens with limited genotyping capacity.^1^, ^8^ These limitations, combined with the possible emergence of novel or atypical pathogens, underscore the need for less restrictive/constrained methods with improved pathogen detection capacity and sufficient genomic information that could assist in proficient diagnosis and possible outbreak management.^4^, ^9^, ^10^

On the other hand, however, metagenomic next‐generation sequencing (mNGS) has helped to integrate and expand upon numerous benefits of molecular testing and culture‐based techniques.^7^, ^11^, ^12^, ^13^, ^14^ For instance, host‐and pathogen‐derived nucleic acids can be sequenced without a prior knowledge, enabling simultaneous detection of a potentially limitless number of microorganisms with sequence homology to reference sequences.^13^, ^15^, ^16^ In addition, mNGS‐based detection methods offer considerable diagnostic potential because alternative causes can be detected or ruled out with better certainty.^17^, ^18^ Furthermore, mNGS enables pathogen subtyping/genotyping, identification of drug resistance markers, and molecular‐based epidemiological studies.^9^, ^19^, ^20^

Although recent studies have demonstrated the power of mNGS in diagnosing respiratory infection in different parts of the world,^6^, ^9^, ^14^, ^21^ the current data from South Africa/Africa at large on the clinical diagnostic utility of mNGS in SARI are still limited,^22^ especially in comparison to the performance of mRT‐PCR in paediatric patients. Additionally, the increasing need for prompt analysis of generated mNGS data has birthed several user‐friendly, nontechnical, yet sensitive web‐based mNGS bioinformatic analysis tools.^23^, ^24^ Nevertheless, different bioinformatic tools may produce varying results for the same FASTQ file.^24^ Therefore, evaluating the viral detectability of some of these mNGS tools is imperative.

In this study, we report the performance of mNGS with three rapid web‐based bioinformatic tools for detecting respiratory viruses in a cohort of paediatric subject's ≤5 years admitted with SARI in the Free State Province, South Africa. While the pathogen profile of these patients has just been recently reported using a new 21 target mRT‐PCR (QIAstat‐Dx‐ Respiratory SARS‐CoV‐2 panel),^25^ the current study evaluated and further expanded the clinical diagnostic utility of mNGS in children with SARI and established a benchmark for future research in the study settings.

## 2 METHODOLOGY

### 2.1 Study settings and demography

The patients, children ≤5 years of age, admitted with SARI were recruited from Botshabelo Hospital, Pelonomi Hospital, National Hospital, and Universitas Academic Hospital in the Free State Province, South Africa. The World Health Organization (WHO) case definitions for SARI^26^ were implemented.

### 2.2 Ethical considerations

Institutional (number UFS = HSD2019/1129/2910) and Provincial ethical approvals were processed and obtained before commencement of the study. Information document with a clear description of the study as well as permission and consent form in the language of choice was provided for the parent/guardian of the children recruited for the study.

### 2.3 Patient enrollment and sample collection

Patient's enrollment and sample collection are as previously described,^25^ Briefly, a total of 84 children admitted with SARI as per the WHO definition, whose nasopharyngeal swab samples in viral transport media (BD Diagnostics) were collected between December 2020 and August 2021 and had previous mRT‐PCR result, were included in this study.

### 2.4 Sample processing, enrichment, and extraction of RNA viruses

Briefly, 1000 µL of the VTM (BD Diagnostics) was centrifuged at 10 000*g* for 10 min to remove cellular debris. The supernatant was filtered through a 0.22 µm filter to remove the remaining possible host cellular debris and bacteria. The filtrate was treated with a nuclease mixture of 0.1U_L−1 Turbo DNAse (Life Technologies), 0.1U_L−1 RNAse One (Promega) and 1X DNAse buffer (Life Technologies) and incubated at 37°C for 90 min, to remove nonincorporating nucleic acid. Nucleic acid extraction was performed using the PureLink® viral RNA/DNA Mini Kit (Thermofisher Scientific) following the manufacturer's instructions but without using carrier RNA. Viral‐extracted RNA was eluted in 50 µL of DNase/RNase‐free water.

### 2.5 Pathogen analysis using mRT‐PCR

Pathogen profiling in the samples collected was performed as previously described,^25^ using QIAstat‐Dx Respiratory‐SARS‐CoV‐2 Panel (Qiagen). The panel detects, generates cycle threshold (Ct) value, and differentiates nucleic acid from severe acute respiratory syndrome coronavirus 2 (SARS‐CoV‐2) and other 20 respiratory pathogens. The assay was performed as per the manufacturer's instructions.

### 2.6 DNase treatment, ribosomal RNA (rRNA) depletion and purification

The extracted RNA was quantified using the Qubit RNA High Sensitivity (HS) Assay Kit (Thermofisher Scientific) with the Qubit® 3.0 Fluorometer (Thermofisher Scientific) as per the manufacturer's instructions. The quantified RNA was treated with TURBO DNA‐free™ reagents (Thermofisher Scientific) according to the manufacturer's instructions. The DNase‐treated samples were purified using the RNeasy Mini Kit (Qiagen) as per the manufacturer's instructions. Depletion of rRNA was performed to decrease the human, mouse, and rat rRNA by using NEBNext rRNA depletion kit (New England Biolabs) according to the manufacturer's instructions.

### 2.7 Reverse transcription and whole transcriptome amplification

For library preparation purposes, isolated viral RNA was reverse‐transcribed to generate complementary DNA (cDNA). For cDNA synthesis and random amplification, a QIASeq FX Single Cell RNA Library Preparation Kit (Qiagen) was used as per the manufacturer's instructions. Amplified cDNA was subsequently quantified using the Qubit™ 1X dsDNA HS Assay Kits with the Qubit 3.0 fluorometer (Thermofisher Scientific) as per the manufacturer's instructions and normalized to the required 100–1000 ng/µL input DNA in 10 µL volume.

### 2.8 Library preparation and next‐generation sequencing

Libraries were prepared using the QIASeq FX Single Cell RNA Library Kit (Qiagen) according to the manufacturer's instructions. The DNA libraries were generated and analysed for average fragment size distribution using the Agilent 2100 Bioanalyzer (Agilent Technologies). Metagenomic sequencing was performed on the Illumina MiSeq system with the reagent kit v3 (Illumina) for 600 cycles to generate 2 × 250 bp paired‐end reads.

### 2.9 Bioinformatic analysis for pathogen detection

The generated paired‐end reads were analysed using three mNGS analysis tools, including the Genome detective (https://www.genomedetective.com/); an automated web system for virus identification from high‐throughput sequencing data,^27^ One codex analysis platform, where detected organisms were displayed as high abundance (HA) (>25% of the sample), medium abundance (5%–25% of the sample), and low abundance (0.5%–5% of the sample),^28^ and the Twist Respiratory Virus Research Panel (TRVRP). The TRVRP is a culture‐free, hybrid capture enrichment workflow embedded as a sub analysis panel in One Codex analysis platform and is capable of detecting 29 common human respiratory viruses simultaneously. Briefly, for the TRVRP, raw sequencing reads (FASTQ) were aligned to each individual viral reference and viruses were reported as “detected” if ≥20% of the genome was observed with an overall average of ≥10x depth. Respiratory viruses were reported as “indeterminate” if there is coverage of ≥5% of the genome, but the data falls short of that required for a “detected” call. All other viruses were noted as “not detected” but the corresponding number of reads and genome coverage are still made available. A BLAST analysis of matching sequences was further performed for some of the detected pathogens (those with very few reads/genomes coverage) to exclude false positive results. Characteristic details of the mNGS analysis tools used in this study are described in Table S1.

### 2.10 Inclusion of negative control

To evaluate for contamination, a no template control (nuclease free water) was subjected through the same mNGS workflow as the samples to assess the presence of cross‐contamination or kitome contamination from reagents.

### 2.11 Statistical analysis

Raw data were calculated using Microsoft Excel 365 version 2206, and results are presented as simple percentages, mean, and standard deviations of replicate determinations.

## 3 RESULTS

### 3.1 Cohort clinical and demographic characteristics

The detailed demographic and clinical characteristics of the participants enrolled in this study are as previously described.^25^ Demographically, 34 (40.4%) were females, while 50 (59.6%) were males. The ages of the participants ranged from 0 to 60 months, with most participants, 57 (67.8%) aged 0–24 months. Also, 35 (41.6%) of the participants required oxygenation, and 17 (20%) were HIV infected, 24 (28.5%) had chest indrawing, 9 (10.7%) had feeding difficulty, 3 (3.5%) were asthmatic, and none of them required the need for admission into the intensive care unit.

### 3.2 Viral detection by mNGS

**Figure 1:**
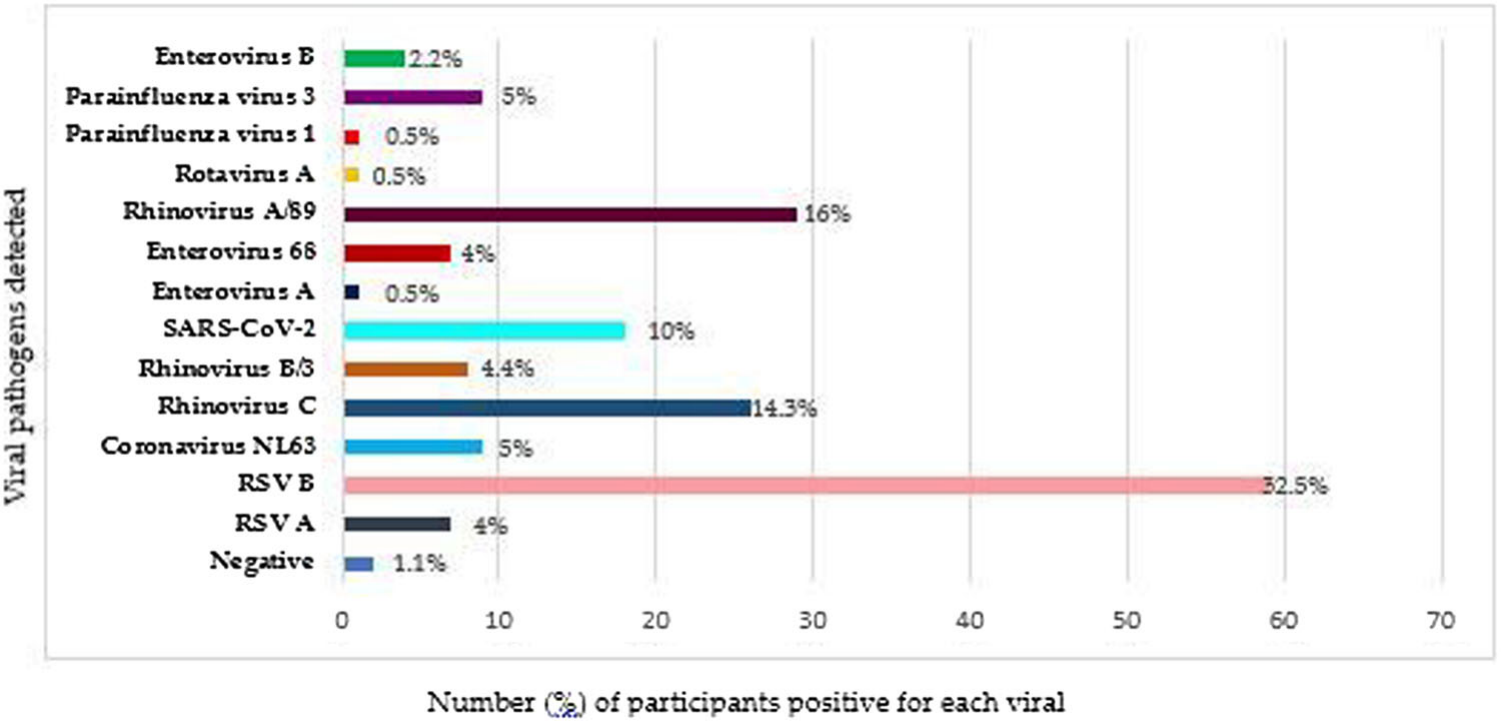
Clustered bar chart of the percentage of patients positive for the detected respiratory viruses via mNGS using the combined results of the three analysis tools. mNGS, metagenomics next‐generation sequencing.

Sequencing of the cDNA libraries from all samples generated an average of 211323 reads after quality trimming and filtering (as per genome detective). Based on the results of the three analysis tools, mNGS detected viral pathogen/s in 82 (97.6%) of 84 participants included in the study. Further insight into the results revealed that respiratory syncytial virus (RSV) B (32.5%), human rhinovirus (HRV) C (14.3%), HRV A/89 (16%), and SARS‐CoV‐2 (10%) were the most frequent pathogens detected (Figure 1 and Table S2). Of the 82 positive samples, single, double, triple, quadruple, and quintuple viral co‐infections were detected in 33% (*n* = 27), 32% (*n* = 26), 19.5% (*n* = 16), 13.4% (*n* = 11), and 2.4% (*n* = 2) patients, respectively (Table S2). The highest genome coverages were mostly for RSV. The detailed mNGS results based on the three analysis tools is presented in Table S2, and additional detection spectrum of mNGS is shown in Table 1.

**Table 1:** Detection spectrum of the viral pathogens using mNGS versus mRT‐PCR.

Detection spectrum	Number of patients (%)
Both methods detected exact number of viral pathogens	22 (26.1)
mNGS detected all viral pathogen detected by mRT‐PCR with at least 1 additional.	15 (18)
mRT‐PCR detected all viral pathogen detected by mNGS with at least 1 additional	19 (22.6)
No viral pathogen detected by mRT‐PCR but detected by mNGS	9 (10.7)
No viral pathogen detected by mNGS but detected by mRT‐PCR	1 (1.2)
mNGS detected more viral pathogen but missed at least 1 detected by mRT‐PCR	7 (8.3)
mRT‐PCR detected more viral pathogen but missed at least 1 detected by mNGS	3 (3.5)
Negative in both methods	1 (1.2)
Both detected one or more viruses but missed by the other	6 (7.1)
Both detected totally different viruses	1 (1.2)

Abbreviations: mNGS, metagenomic next‐generation sequencing; mRT‐PCR, multiplex real time polymerase chain reaction.

### 3.3 Viral pathogen detectability of multiple mNGS analysis tools

**Figure 2:**
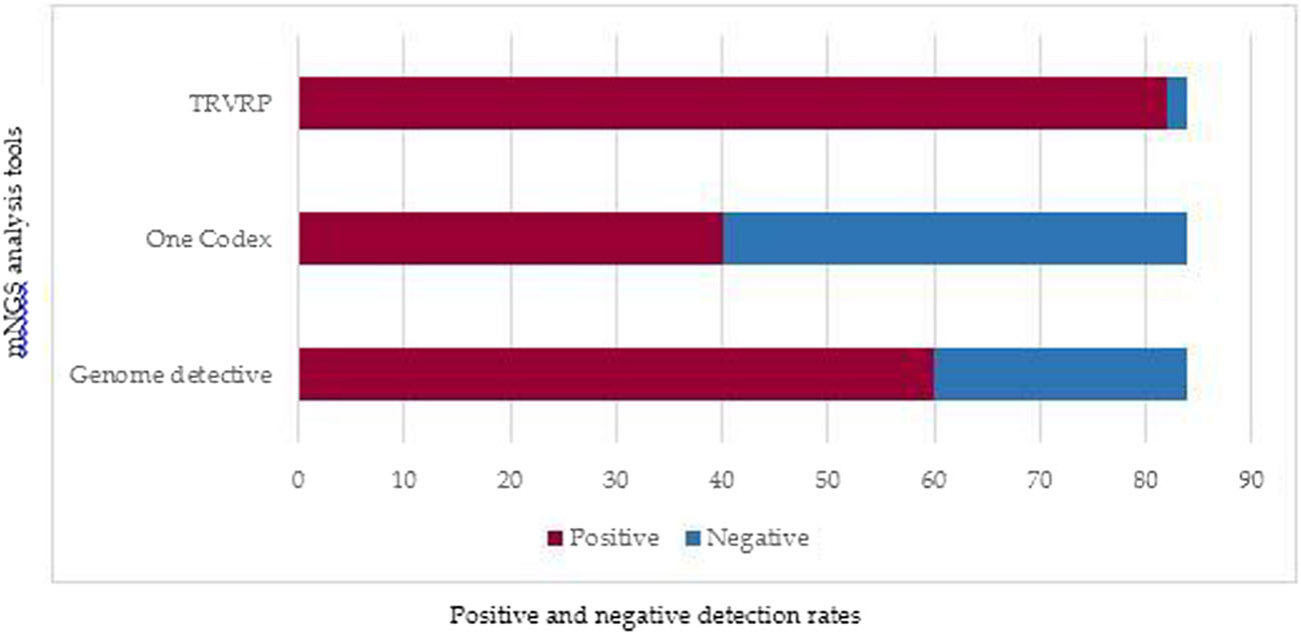
Viral pathogen detectability of the three mNGS analysis tools; Genome Detective, One Codex, and TRVRP (Twist Respiratory Virus Research Panel). mNGS, metagenomics next‐generation sequencing.

The use of multiple analysis tool increased the viral detection rate of mNGS. Using the detection of at least one viral respiratory pathogen as a positivity ratio, TRVRP detected more viral co‐infections, detected more viral pathogen present at low abundance (Ct value ≥35) and had the highest positive detection rate (98%) compared to One Codex (48%) and Genome detective (71%) (Figure 2 and Table 2).

**Table 2:** Viral pathogen detection spectrum of the three mNGS analysis tools (Genome Detective, One Codex, and TRVRP [Twist Respiratory Virus Research Panel]).

Detection spectrum	Number of subjects
Instances where similar number of pathogens were detected by the three methods per subjects	12
Instances where the three methods detected different viral pathogens	3
Instances where the three methods had no viral pathogen detected	2
Instances where similar number of pathogens were detected by Genome detective and TRVRP	9
Instances where similar number of pathogens were detected by One Codex and TRVRP per subject.	4
Instances where Genome Detective detected more viral pathogens per subjects	3
Instances where One Codex detected more viral pathogens per subjects	3
Instances where TRVRP detected more viral pathogens per subjects	30
Instances where TRVRP detected at least a viral pathogen where others had no viral detection	18
Instances where Genome Detective detected at least a viral pathogen where others had no viral detection	0
Instances where One Codex detected at least a viral pathogen where others had no viral detection	0
Instances where a pathogen present at very low abundance (as seen by mRT‐PCR Ct value ≥35.0) was detected by Genome Detective	0
Instances where a pathogen present at very low abundance (as seen by mRT‐PCR Ct value ≥35.0) was detected by One Codex	0
Instances where a pathogen present at very low abundance (as seen by mRT‐PCR Ct value ≥35.0) was detected by TRVRP	6

Abbreviations: mNGS, metagenomic next‐generation sequencing; mRT‐PCR, multiplex real time polymerase chain reaction.

### 3.4 Bacteria detection

**Figure 3:**
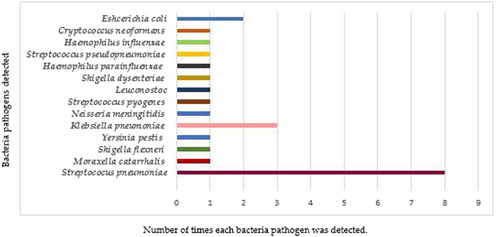
A bar chart of the detected bacteria pathogens using One Codex mNGS analysis platform. mNGS, metagenomics next‐generation sequencing.

Via One Codex Analysis, bacterial co‐infection was detected in 24/84 (28.5%) patients, most of which were *Streptococcus pneumoniae* 8/84 (9.5%), *Klebsiella pneumoniae* 3/84 (3.5%), *Escherichia coli* 2/84 (2.3%), and one each of the remaining bacteria pathogens shown in Figure 3. Complete data on reads and abundance is shown in Table S2. The remaining two analysis tools (Genome Detective and TRVRP) only detects viral sequences.

### 3.5 Detection of nonrespiratory/nonhuman viruses and bacteriophages

In addition to the detected common respiratory viruses. Reads for other viruses constituting the respiratory virome were detected. This includes human endogenous retrovirus k113, plant, fungal, animal, and bacteriophages that primarily infect bacteria known to colonize the upper respiratory tract (Table S3).

### 3.6 Evaluation of the negative control

No respiratory or human‐pathogenic viral reads were detected in the negative control (nuclease free water) across the three mNGS analysis tools. However, One Codex analysis tool detected some bacteria reads in the negative control (Table S4). These bacteria reads at low abundance were filtered out of the actual results during bacteria analysis.

## 4 DISCUSSION

The respiratory tract is a target of many human viruses, especially RNA viruses. The current molecular techniques for detecting respiratory viruses are largely targeted‐dependent tests, which detect a limited number of viruses. Therefore, it often leaves clinicians with negative results and the question of alternative aetiology for which testing was not done.^11^ The untargeted nature of mNGS allows the query of theoretically unlimited number of pathogens in parallel, resulting in increased viral detection and a higher positivity rate. The detected percentage average reads (4.8%) in this study was in the same range (2%) for similar sample types previously reported,^29^ but higher than the 0.32% and 0.05% reported by Romero‐espinoza et al.,^30^ and Yang et al.,^31^ respectively. Also, the observation regarding the performance of mNGS (97.6%) viral pathogen detection rate in this study is similar to those previously reported by Graf and colleagues (91.8%) ^9^ but higher than the 43%, 32.6%, and 63% reported by Thorbun et al.,^19^ Madi et al.,^29^ and Graf et al.,^9^ respectively. The variability in the reported detection rate may be due to several factors that tend to influence the amount of virus in each sample. These factors include location, sampling technique, and preanalytical sample preparation. All of which may greatly impact the relative amount of the viral genome available to be sequenced.^32^ Enrichment of viral nucleic acids from clinical samples before sequencing as adopted in this study is thus crucial.

The need for immediate molecular subtyping of viral species continues to garner interest; this is due to the several advantages of subtyping, including identification of genotypic markers of drug resistance or pathogenicity. In this study, relevant information derived from mNGS typing included the following: (i) almost 80% of HRV in this study belonged to HRV A and the more pathogenic species C; (ii) RSV‐B was more prevalent than RSV‐A, which may be relevant, as strain‐specific differences in pathogenicity have been suggested^33^ or may be due to infection prevalence of RSV‐B in the community; (iii) detection of Enterovirus 68 subtype in 4% of the patients; a pathogen once linked to more severe and fatal respiratory illnesses than other enterovirus genotypes^34^; (iv) detection of Enterovirus B subtype in 4 patients; a pathogen previously linked with viral meningitis in children and adults.^35^ The specific identification of these viruses by mNGS (especially of HRV and Enterovirus with sequence similarities in the commonly used conserved targets for molecular assays) can have implications for the supportive management of patients and can become more significant when specific antiviral drugs become available.^36^ Moreover, as genotype‐phenotype correlations become better understood, genotypic strain characterization will gain importance and may further facilitate epidemiologic investigations or studies of vaccine effectiveness.^9^, ^37^, ^38^ Furthermore, the uncovered respiratory virome (constituting bacteriophages, plant virus, animal virus, human endogenous retrovirus k113 and algae viruses) in each participant in this study reflects the transient or permanent colonization of nonhuman viruses in the respiratory milieu. While some of these nonhuman viruses were sparsely detected, reads for bacteriophages were consistently detected at varying amount in all participants. This support previous reports on HA of bacteriophages in the respiratory tract with the potential to prime and modulate host immune responses in children.^39^, ^40^ Additionally, mNGS further enabled the simultaneous detection of bacteria co‐infection in 24 (28.5%) patients despite enrichment for RNA viruses. This phenomenon is due to the significantly known high representation of bacterial DNA including pathogenic and commensals bacteria which may be present in clinical samples, and often outnumbers viral nucleic acids. Despite strict sample enrichments for viral RNA, present bacterial nucleic material is still often carried over and may be amplified during the nonspecific whole transcriptome amplification step. Nonetheless, the ability of mNGS to concurrently detect pathogenic bacterial nucleic material in a single test despite sample enrichment for RNA viruses as shown in this study further points to the power of syndromic testing of mNGS^41^ which could be harnessed and may futuristically serve as an add‐on test to several culture and PCR‐based tests.^9^

Recently, mNGS reports have been benchmarked against traditional methods such as mRT‐PCR or culture to further establish the utility of mNGS in clinical diagnostics. Compared with the 97.6% detectability rate in this study by mNGS, mRT‐PCR results in the same participants yielded 88% positivity detection rate.^25^ Besides, mNGS identified more viral pathogens (179 vs. 154) than the mRT‐PCR. Further, mNGS identified at least a viral pathogen in 9 of 10 patients who were negative to the 21 targeted pathogens by mRT‐PCR.^25^ These results are in line with the previous findings, confirming that mNGS detection can improve the positive rate of pathogen detection. A study by Lewandowska^32^ and colleagues confirmed a viral aetiology of infection by mNGS approach in four patients despite initial negative results in specific multiplex PCR. Another study by Li et al.^42^ performed NGS detection on BALF samples from 32 patients, and the positive detection rate of NGS reached 100%. Zhang et al.^43^ also reported that the total positive rate detected by mNGS method (91.1%) in the diagnosis of acute respiratory distress syndrome was significantly higher than that detected by the culture method (62.2%). Similarly, Huang and colleagues compared NGS with traditional pathogen detection methods in diagnosing peripheral pulmonary infectious lesions and reported a detection accuracy of 86.25% vs 43.75% for mNGS and traditional detection methods, respectively.^44^ Despite the reported increased favorable detection rates reported for mNGS in this study and other studies, lower performances of mNGS compared to PCR has been documented. A study by Thorburn et al.^19^ reported 43% versus 54% positivity rates for NGS and RT‐PCR, respectively. Similarly, Madi et al.^29^ reported 75.6% versus 32.6% positivity rates for mRT‐PCR and mNGS, respectively. However, both studies highlighted the several advantages of mNGS over the mRT‐PCR and suggested that lower abundance of the viruses detected by RT‐PCR could have resulted in a lower detection rate by mNGS.

Furthermore, corresponding with the previously reported data from the mRT‐PCR assay in the same patients,^25^ RSV and HRV were the most predominant viral pathogens detected in the respiratory samples by the mNGS‐based approach. This observation agrees with others who showed that RSV and HRV are the most prevalent viruses in children with SARI.^4^, ^8^, ^45^, ^46^ Notably, the mRT‐PCR detected 32 cases of SARS‐CoV‐2 in the study participants.^25^ On the contrary, mNGS only detected 18 of the 32 SARS‐CoV‐2 cases. This comparatively lower detectability rate is not surprising, considering that most of the SARS‐CoV‐2 cases detected by mRT‐PCR had Ct values mostly ≥33.^25^ Such higher Ct values (correlative of low viral load) have been previously reported to impact viral detection by mNGS.^19^, ^29^ Similar to the mRT‐PCR result from the same participants, there was no detection of the seasonal influenza virus by mNGS. This could be attributed to the sampling/study period where zero influenza detection was reported in the Free State Province from week 50 (December) of 2020 to week 34 (August) of 2021 as per the National weekly respiratory pathogen surveillance report by the National Institute for Communicable Diseases, (NICD) South Africa (https://www.nicd.ac.za/diseases-a-z-index/disease-index-covid-19/surveillance-reports/weekly-respiratory-pathogens-surveillance-report-week/) (a summary of the weekly influenza surveillance data per province for the study period is shown in Table S5). Contrary to the nondetection of influenza, nationally, an outbreak of RSV outside of its season followed by its sporadic detection^47^ and waves of SARS‐CoV‐2 transmission (https://www.nicd.ac.za/wp-content/uploads/2021/11/Proposed-definition-of-COVID-19-wave-in-South-Africa.pdf) were documented during the study period. This observed phenomenon is, however, attributed to the nonpharmaceutical interventions implemented against COVID‐19 and was also corroborated in a previous study.^25^

Also, the nondetection of adenovirus by the mNGS approach may be due to the initial DNAse step outlined in the methodology. Additionally, mNGS in this study identified several viral co‐infection rates correlative with the mRT‐PCR result. Viral co‐infections detected by the mRT‐PCR in the same participants ranged from double to quadruple,^25^ while mNGS detected double to quintuple. However, the association of co‐infection to disease severity or clinical outcome could not be established due to the unavailability of information on death history, if any, or exacerbated clinical course. Of interest, of the two patients with no viral disease‐related pathogen detected by mNGS, one had a viral pathogen detected, and the other was equally missed by mRT‐PCR. Hypothetically, the viral aetiology could be present at a low abundance below the sensitivity of the mNGS, could be a novel virus not detected in the bioinformatic analysis, a DNA virus not targeted in the workflow, or rather an aetiology other than of viral origin as seen by the detection of *Escherichia coli* in the patient (Z44) missed by both methods.

The incremental value of multiple rapid, user‐friendly mNGS analysis tools has been previously reported.^23^, ^34^ Currently, there are no gold standard mNGS analysis tools, and different methods can provide varying outcomes for the same FASTQ file due to differences in the databases used by each tool.^24^, ^48^ In this study, a higher detection rate was noted for the TRVRP; it detected higher co‐infection rate, detected more viral pathogen present at low abundance and reported reads for at least one viral pathogen in 98% of the patients compared to 71% and 48% by Genome Detective and One Codex analysis tools, respectively. The detection rate exhibited by the TRVRP may be due to the use of the GenBank database. However, unlike Genome detective and One Codex analysis tools, any untargeted viral pathogen will not be detected. Notably, the number of detected reads and genome coverage for each viral pathogen varied significantly between the three mNGS analysis tools, explaining in part the variations in detection limits of each tool.^23^ Although study‐specific cut‐off points for defining positive detection were defined for the three mNGS analysis tools. Nonetheless, this study detected all pathogens well above the cut‐off points. While the use of multiple mNGS analysis tools as described may bolster mNGS results, further improvements/benchmarking of these analysis tools is required and upgrading of the data library to overcome deficiencies of the individual methods or databases is equally important.

Finally, the case of a participant (patient A25) further highlights the complex microbial basis of SARI. This female patient, aged 4 years and 5 months old, was admitted with SARI (upper respiratory infection of pneumonia and difficulty in breathing) and had a nasopharyngeal sample collected on 01/01/2021. The patient was HIV‐negative and did not need intensive care or oxygen. The pathogen detection using mRT‐PCR was negative for all the targeted pathogens, and only HRV was detected via mNGS using TRVRP at 0.44% genome coverage. However, reads for *Neisseria meningitidis* was detected via One Codex at a HA of 97.91%. The history of meningococcal vaccination is unknown in the child. However, meningococcal vaccination is not included in the South Africa vaccine schedule and is only available for infants and adolescents in the private sector. While latent colonization has been reported in children,^49^ a detection at 97.91% abundance may suggest replication/active infection and could be the main factor for symptoms exhibited.

Despite the apparent advantages of mNGS in viral pathogen detection, there are still some gaps to bridge. For instance, the observed low sensitivity of mNGS for the detection of samples with low viral load, especially of SARS‐CoV‐2, as seen in this study impedes the unequivocal reliability of this method. As such, the requirement for improved sample preparation, for low biomass pathogens and optimized analysis tools, is still imperative ^10^ More so, the detection of viral reads may not translate to the presence of replicable viruses. Nonetheless, in some cases, viral infection may be limited to a specific area of a tissue or part of susceptible cells. As such, detection of a single or very few viral reads may be indicative of viral infection.^50^ As seen in this study, there were many correlative viral detections by mRT‐PCR and mNGS. However, in some cases, the viral reads from mNGS analysis were comparatively low, despite being a true positive. Consequently, it raises the question of what limit of detection is applicable/should be implemented to enable intra/inter‐laboratory comparisons.^7^, ^23^, ^50^

A notable limitation of this study is the strict enrichment for RNA viruses, which makes it impossible to detect adenovirus (as detected by the mRT‐PCR) or account for other co‐infecting DNA viruses using mNGS. Consequently, further studies may be required to evaluate the detection of DNA viruses using mNGS compared to mRT‐PCR. Additional limitation is the absence of the inclusion of positive control which could have been used in the evaluation of false negatives.

## 5 CONCLUSION

This study further reiterates that mNGS‐based detection holds promise as a diagnostic tool in severe paediatric infectious viral respiratory diseases, enabling the unbiased detection and molecular typing of the viral respiratory pathogens with simultaneous elucidation of the respiratory virome. The study presented much‐needed information on the clinical utility of mNGS in SARI in the study setting where limited data on usability currently exist. Although further improvement of mNGS workflow (wet and dry lab onuses) may be required, mNGS can be used to supplement PCR‐based tests, especially in cases where traditional methods have yielded no result or instances where symptoms are severe, and the detected pathogen is not truly reflective of the symptoms presented. Imperatively, future studies where mNGS fails to yield a positive result may benefit from increased sequencing depth that could even obviate the need for enrichment steps, target of both DNA and RNA virus and differential sampling to include lower respiratory samples. Furthermore, the power of mNGS in syndromic testing and the significant incremental value of multiple user‐friendly mNGS analysis tools was strongly demonstrated. Moving on, further implementation of trials to benchmark mNGS analysis tools and ensure comparable results and upgrade of the different analysis tools are required. Excitingly, sequencing costs are beginning to decline, and as evidence of clinical utility accumulates, the use and experience with this approach will probably become more widespread over time, stimulating the continuous development and optimization of mNGS for clinical diagnostic use.

## AUTHOR CONTRIBUTIONS

*Project conceptualization, supervision, and administration*: Martin M Nyaga, Sabiu Saheed, and Ayodeji E Ogunbayo. *Sample delivery*: Ayodeji E Ogunbayo and Milton T Mogotsi. *Data curation*: Ayodeji E Ogunbayo, and Hlengiwe Sondlane. *Laboratory investigation*: Ayodeji E Ogunbayo, Milton T Mogotsi, Hlengiwe Sondlane, and Martin M Nyaga. *Data analysis and presentation*: Ayodeji E Ogunbayo. *Original draft*: Ayodeji E Ogunbayo. *Writing—review and editing*: all co‐authors. *Funding*: Martin M Nyaga.

## CONFLICT OF INTEREST STATEMENT

The authors declare no conflicts of interest.

## ETHICS STATEMENT

The study was conducted in accordance with the Declaration of Helsinki and approved by the Health Science Research Ethics Committee (HSREC) of the University of the Free State, South Africa (Approval no UFS‐HSD2019/1129/2910 and approval date of 24 October 2019). The study was also approved by the Free State Department of Health.

## Supporting information

Supporting information.

## Data Availability

The data presented in this study are available in Tables S2–S4.
