# Supplementary material for: Metagenomics characterization of respiratory viral RNA pathogens in children under five years with severe acute respiratory infection in the Free State, South Africa
Source: J Med Virol. 2023 Apr 27;95(5):e28753. doi: 10.1002/jmv.28753 (PMC10952945; doi:10.1002/jmv.28753)
Supplement: Supplementary file 1 — Supporting information. [file JMV-95-0-s001.docx]

| **Classification tool** | **Genome detective v 2.40** | **One Codex Database 2021** | **Twist Respiratory Viral Research Panel** |
| --- | --- | --- | --- |
| **Clinical usage by participant** | Patient care **^a^** | Experimental | Research |
| **In-house/ commercially available** | Commercial | Commercial | Commercial |
| **Local/web-based** | Web based | Web based | Web based |
| ***De novo* assembly** | Yes | No | N/A |
| **Alignment of NT/ AA** | NT/AA | NT | Aligns to targeted sequences |
| **Database used by participant viral/bacterial (version)** | Viruses; based on RefSeq (filtering: Swissprot Uniref 90) v2018- | Bacteria, viruses, fungi, archaea, protozoa, One Codex DB v2019 | RefSeq and GenBank database |
| **Paired reads as input option** | Yes | Yes | Yes |
| **Trimming and QC tools** | Trimmomatic and FastQC | Cutadapt | N/A |
| **Exclusion of human reads** | Yes | Yes | N/A |
| **Output type** | Web interface, interactive, and Excel | Web interface, interactive,  PDF and Excel | PDF |
| **Visualization of genome coverage** | Yes | No | Yes |
| **Computational time for analysis per sample (CPU/RAM)** | ~10 nm (web-based) | 35-40 nm (web-based) | ~10 nm (web-based) |
| **Cut-off for defining positive result used** | ≥3 regions, distributed | 1 read | ≥20% of genome |
| **Confirmatory analysis required for clinical reporting** | Undisclosed | Not required | Undisclosed |
| **Adapted study specific cut off for defining a viral pathogen as detected.** | ≥3 reads or ≥3 regions, distributed | ≥1 read | ≥0.5% genome coverage |

**Supplementary Table 1:** Clinical use, classification, and characteristics of the three mNGS analysis tools [24]

AA; amino acid, NT; nucleotide, N/A; Not applicable, a; withing the scope of accreditation

**Supplementary Table 2:** Pathogens detected using three metagenomics next-generation sequencing analysis tools

| **Sample Record No** | **Genome Detective**  **Total Reads/viral reads%** | **Genome detective**  **Detected pathogen/Reads/Genome Coverage** | **One Codex**  **Detected pathogen/Abundance/Reads** | **Twist Respiratory Viral Research Panel**  **Detected pathogen/Genome Coverage/Identity** |
| --- | --- | --- | --- | --- |
| A301 | 119726/5% | Human orthopneumovirus (Subgroup B) **4/1.5%** | -------------------------------------- | Human orthopneumovirus **3.74%/95.90%**  Human rhinovirus C **0.85%/93.33%** |
| Z55 | 284596/4% | Human orthopneumovirus (Subgroup B) **1278/84.4%**  Rhinovirus C **24/8.9%**  Human respirovirus 3 **2/0.9%** | ---------------------------------------- | Human orthopneumovirus **93.09%/96.70%**  Human parainfluenza virus 3 **0.75%/95.06%**  SARS-CoV-2 **0.73%/99.32%**  Human rhinovirus C **2.8%/99%** |
| Z11 | 108212/3% | Human orthopneumovirus (Subgroup B) **568/36.7%** | ------------------------------------ | Human orthopneumovirus **13.32%/96.87%**  Human rhinovirus C **1.32%/95.74%** |
| A29 | 67664/6% | Rhinovirus A **42/14.7%** | *Streptococcus pneumoniae* **HA/76**  Rhinovirus A  **MA/45**  Rhinovirus C **LA/2** | Human orthopneumovirus **1.10%/94.64%**  Human rhinovirus 89 **1.59%/92.4%**  Human rhinovirus C **1.75%/95.9%**  SARS-CoV-2 **0.57%/100%** |
| A34 | 227390/5% | Rhinovirus C **3144/34.3%**  Human orthopneumovirus (Subgroup A) **30/5.5%** | RSV **HA/790**  Rhinovirus C **MA/74** | RSV A **87.54%/96.63%**  Rhinovirus C **0.87%/100%** |
| Z26 | 215998/4% | Rhinovirus A **14/4.6%** | Rhinovirus A **HA/12** | Human rhinovirus 89 **5.29%/95.95%** |
| Z58 | 96522/4% | Rhinovirus C **23452/43%**  Human orthopneumovirus (Subgroup A) **600/48%** | Rhinovirus C **MA/16**  RSV **HA/655** | Rhinovirus C **7.85%/97%**  RSV A **88.5%/98.9%** |
| Z51 | 128324/8% | ------------------- | RSV **HA/84**  *Streptococcus pneumoniae* **MA/101**  Rhinovirus C **MA/18** | Human orthopneumovirus **30.14%/96.81%** |
| Z38 | 138188/5% | Human respirovirus 3 **13/2.6%** | RSV **HA/112**  Rhinovirus A **MA/12**  Rhinovirus C **MA/4**, Human respirovirus 3 **MA/17**, Human respirovirus 1 **LA/4** | Human orthopneumovirus **34.17%/96.95%**  Human parainfluenza virus 1 **1.72%/95.91%**  Human parainfluenza virus 3 **5.45%/95.02%**  Human rhinovirus 89 **1.48%/94.84%** |
| Z7 | 257964/3% | ------------------ | ------------------------------------ | ------------------------------------ |
| Z34 | 141204/6% | Human orthopneumovirus (Subgroup B) **8/1.4%** | ------------------------------------- | Human orthopneumovirus **17.24%/96.53%**  Human rhinovirus C **0.58%/98.75%**  Human enterovirus 68 **21.3%/100%** |
| Z32 | 207416/5% | ----------------------- | ----------------------- | Human orthopneumovirus **15.79%/98.35%** |
| Z49 | 73486/2% | Human orthopneumovirus (Subgroup B) **865/72.8%** | RSV **HA/2783** | Human orthopneumovirus **90.72%/96.66%**  SARS-CoV-2 **0.47%/99.34%** |
| Z75 | 127376/3% | ----------------------- | -------------------------------------- | SARS-CoV-2 **1.03%/99.54%** |
| Z27 | 169354/4% | Human orthopneumovirus (Subgroup A) **91/23%** | RSV **MA/21** | RSV A **66.99%/99%** |
| Z44 | 111650/2% | ------------------------- | *Escherichia coli* **HA/123** | ---------------------------- |
| Z37 | 192552/7% | Human orthopneumovirus (Subgroup A) **170/20.4%**  Enterovirus A 209/30.4% | RSV **HA/334**  Enterovirus A **MA/9** | RSV A **68.25%/95.93%**  SARS-CoV-2 **0.44%/100%**  Human enterovirus 68 **0.46%/100%** |
| Z67 | 192016/4% | Rhinovirus C **10/4.8%**  Human respirovirus 3 **6/1.6%** | Rhinovirus C **MA/1**  Human respirovirus 3 **MA/16** | Human parainfluenza virus 3 **2.74%/95.76%**  Human rhinovirus 89 **1.54%/92.74%** |
| W43 | 299216/3% | --------------------- | ---------------------------------------- | Human orthopneumovirus **7.38%/97.40%** |
| Z9 | 150700/5% | -------------------------- | *Streptococcus pneumonia* **HA/235** | Human orthopneumovirus **1.12%/98.03%** |
| Z23 | 105086/2% | Human orthopneumovirus (Subgroup B) **3/1.9%** | RSV **MA/21**  *Streptococcus pneumoniae* **LA/61**  Coronavirus NL63 **LA/2** | Human orthopneumovirus **90.04%/96.66%**  Human coronavirus NL63 **0.60%/98.33%** |
| Z15 | 388568/8% | Human orthopneumovirus (Subgroup B) **32/11.5%** | ------------------------------------- | Human orthopneumovirus **4.74%/97.27%**  Rhinovirus 3 **0.47%/100%**  SARS-CoV-2 **0.67%/99.60%** |
| W6 | 197070/6% | Rhinovirus C **178/23.8%** | RSV **MA/24** | RSV A **15.56%/96.39%**  Human rhinovirus C **0.72%/96.08%** |
| W26 | 286194/7% | SARS-CoV-2 **6/0.8%** | SARS-CoV-2 **LA/8**  Human orthopneumovirus **LA/12**  Rhinovirus **LA/6** | Human orthopneumovirus **0.38%/98.28%**  Human rhinovirus 89 **0.98%/92.89%**  SARS-CoV-2 **0.41%/99.19%** |
| W33 | 107188/4% | ------------------------- | ------------------------------- | Human orthopneumovirus **1.52%/96.97%**  Human rhinovirus 89 **0.89%/92.19%** |
| W12 | 272120/6% | Human orthopneumovirus (Subgroup B) **92/21.8%**  Rhinovirus C **230/19.3** | Rhinovirus C **HA/9**  RSV **MA/146** | Human orthopneumovirus **27.66%/96.62%**  Human coronavirus NL63 **0.50%/98.54%**  Human rhinovirus C **0.56%/100%**  Human enterovirus 68 **0.87%/99.83%** |
| W3 | 132868/6% | Rhinovirus C **23/6.9%** | --------------------------------- | Human rhinovirus 89 **0.89%/92.19%**  Human orthopneumovirus **1.60%/97.52%**  Human enterovirus 68 **0.83%/100%** |
| W24 | 541198/3% | Rhinovirus C **856/39.2%**  Human orthopneumovirus (Subgroup B) **2/1.1%** | --------------------------- | Human orthopneumovirus **4.85%/96.70%** |
| W43 | 165243/4% | ---------------------------- | ------------------------------ | Human orthopneumovirus **7.38%/97.40%** |
| Z35 | 81186/6% | Human orthopneumovirus (Subgroup B) **647/54.2**  SARS-CoV-2 **12/0.5%** | RSV **HA/1004** | Human orthopneumovirus **63.31%/96.23%**  SARS-CoV-2 **0.86%/98.20%** |
| Z45 | 206928/8% | Human orthopneumovirus (Subgroup B) **82/17.2%** | -------------------------- | Human orthopneumovirus **26.60%/97.21%**  Human coronavirus NL63 **6.01%/98.75%** |
| W16 | 302482/5% | Rhinovirus C **124/6.8%** | *Moraxella catarrhalis* **LA/3** | Human orthopneumovirus **0.64%/98.98%**  Human rhinovirus **89 2.34%/91.14%** |
| W34 | 186668/6% | Rhinovirus A **12/7.6%** | ---------------------------- | Rhinovirus 89 **5.12%/94.88%** |
| W41 | 180338/4% | Rhinovirus B **35/15.8%** | *Streptococcus pneumoniae* **MA/35** | Human rhinovirus 89 **0.63%/97.73%**  Human rhinovirus 3 **4.11%/93.86%** |
| W40 | 132672/3% | ---------------------------------- | ----------------------------- | Human orthopneumovirus **0.79%/96.69%** |
| W44 | 151444/6% | Rhinovirus C **608/21.3%**  Human coronavirus NL63 **50/7.2%**  Human orthopneumovirus (Subgroup B) **9/4.6%** | Rhinovirus A **HA/859**  Rhinovirus C **HA/45**  RSV **LA/14** | Human coronavirus NL63 **12.19%/98.37%**  Human orthopneumovirus **6.88%/97.33%**  Human rhinovirus 89 **4.03%/91.59%**  Human rhinovirus C **1.65%/87.18%** |
| W42 | 242804/4% | ------------------------------------ | *Moraxella catarrhalis* **HA/73** | Human rhinovirus C **1.82%/89.23%** |
| W27 | 251412/4% | Rhinovirus A **16/7%** | Rhinovirus A **HA/20** | Human rhinovirus 89 **2.10%/96.42%** |
| W19 | 632812/2% | Human orthopneumovirus (Subgroup B) **4448/95.8%** | ------------------------------------------- | Human orthopneumovirus **97.42%/96.93%** |
| W38 | 219696/4% | Rhinovirus C **5/3.3%** | RSV **MA/37**  Human coronavirus NL63 **MA/44** | Human orthopneumovirus **15.59%/96.62%**  Human coronavirus NL63 **11.44%/98.27%**  Human Rhinovirus 3 **2.32%/94.21%** |
| W32 | 159146/2% | Human orthopneumovirus (Subgroup B) **25/4.4%** | RSV **MA/75**  Rhinovirus A **LA/3** | Human orthopneumovirus **29.06%/96/90%** |
| W22 | 131012/3% | --------------------------- | RSV **LA/7**  *Klebsiella pneumoniae* **HA/78** | Human orthopneumovirus **3.88%/96.96%** |
| W31 | 86624/6% | Rhinovirus A **122/19.6%**  Human orthopneumovirus (Subgroup B) **2/1.4%** | Rhinovirus A **HA/112**  RSV **LA/22**  *Shigella flexneri* **MA/14** | Human orthopneumovirus **11.14%/96.63%**  Human rhinovirus 89 **3.38%/95.55%**  SARS-CoV-2 **0.54%/100%** |
| W10 | 497290/9% | Human coronavirus NL63 **1279/58.4%**  Human orthopneumovirus (Subgroup B) **8/3%** | Coronavirus NL63 **HA/2233** | Human Coronavirus NL63 **75.77%/98.21%**  Human orthopneumovirus **6.50%/96.41%** |
| A39 | 470420/3% | ------------------------------ | ---------------------------------- | Human orthopneumovirus **0.89%/97.47%** |
| A28 | 155742/3% | ---------------------------- | *Klebsiella pneumoniae* **LA/2.34** | Human orthopneumovirus **0.64%/96.94%** |
| Z24 | 329486/5% | ------------------------------ | ------------------------------- | Human orthopneumovirus **0.41%/93.55%**  Human rhinovirus 89 **1.89%/95.52%** |
| A25 | 133576/4% | ---------------------------- | *Neisseria meningitidis* **HA/19** | Human rhinovirus 3 **0.44%/100%** |
| A33 | 572190/8% | -------------------------------- | ----------------------------------- | Human orthopneumovirus **3.65%/95.38%**  SARS CoV-2 **2.99%/99.66%** |
| A23 | 180620/4% | Human orthopneumovirus (Subgroup B) **278/22.4%** | ---------------------------------- | Human orthopneumovirus **76.9%/96.67%** |
| A32 | 363296/10% | Rhinovirus A **673/69.9%**  Human orthopneumovirus (Subgroup B) **126/18.6%** | Rhinovirus A **HA/286**  RSV **MA/171** | Human orthopneumovirus **42.99%/96.83%**  Human rhinovirus 89 **3.68%/94.91%**  SARS-CoV-2 **0.20%/100%**  Human rhinovirus C **2.11%/84.23%** |
| A27 | 154530/4% | Rhinovirus A **276/12.6%** | Rhinovirus A **LA/3.78** | Human rhinovirus 89 **2.82%/85.83%**  Human rhinovirus C **2.11%/92.40%** |
| A30 | 125658/3% | Rhinovirus C **57/18%**  Human respirovirus 3 **16/17%** | Rhinovirus C **MA/70** | Human rhinovirus C **23%/98.9%**  Human parainfluenza virus 3 **2.89%/97%** |
| A26 | 170304/5% | Rhinovirus A **56/20%** | Rhinovirus A **HA/70**  Rhinovirus C **LA/12**  Human respirovirus 3 **LA/6** | Human parainfluenza virus 3 **2.66%/96.60%**  Human rhinovirus 89 **3.78%/93.65%**  Human rhinovirus C **2.11%/89.17%** |
| A35 | 127340/3% | Human orthopneumovirus (Subgroup B) **6/0.8%** | ---------------------------------- | Human orthopneumovirus **1.14%/96.81%** |
| A31 | 251098/4% | Human orthopneumovirus (Subgroup B) **6/2.8%** | Enterovirus B **HA/18**  *Streptococcus pyogenes* **MA/12**  RSV **LA/3** | Human orthopneumovirus **3.13%/97.22%**  Human enterovirus 68 **1.66%/86.29%** |
| A38 | 220978/2% | Rhinovirus A **19/4%** | ---------------------------------- | Human orthopneumovirus **0.66%/96.08%**  Human enterovirus 68 **0.42%/100%** |
| Z606 | 146478/5% | Rhinovirus A **2/2.3%** | *Leuconostoc* **HA/231** | Human orthopneumovirus **5.43%/96.06%** |
| A40 | 77958/4% | Rhinovirus C **206/12.8%** | Rhinovirus C **HA/6**, RSV **LA/2**,  *Shigella dysenteriae* **MA/7** | Human orthopneumovirus **2.48%/95.06%** |
| W1 | 141548/6% | Rhinovirus A **4/3.2%**  Human orthopneumovirus (Subgroup B) **6/2.9%** | *Klebsiella pneumoniae* **HA/32** | Human orthopneumovirus **4.17%/96.49%** |
| Z28 | 478500/8% | Rhinovirus A **690/36.4%**  Human orthopneumovirus (Subgroup B) **6/2.5%** | ------------------------------ | Human orthopneumovirus **12.71%/96.86%**  Human rhinovirus 89 **9.55%/96.47%**  SARS-CoV-2 **0.87%/99.23%** |
| Z53 | 90942/4% | Enterovirus B **17/4.3%** | -------------------------------------- | Human parainfluenza virus 3 **9.25%/95.56%** |
| Z76 | 59084/3% | Human orthopneumovirus (Subgroup B) **2/0.9%** | RSV **HA/27**  *Streptococcus pneumoniae* **MA/47** | Human orthopneumovirus **11.84%/95.96%** |
| Z78 | 186888/6% | Human respirovirus 3 **4/0.7%** | ----------------------------------- | Human orthopneumovirus **0.68%/98.08%**  Parainfluenza virus 3 **1.93%/94.06%** |
| Z16 | 280562/4% | ----------------------------- | ------------------------------------- | Human orthopneumovirus 1**.96%/96.36%**  Human rhinovirus 89 **1.79%/85.94%**  Human rhinovirus C **1.76%/100%** |
| W14 | 334740/3% | -------------------------- | Rhinovirus A **HA/1**  Rhinovirus C **MA/3** | Human rhinovirus 89 **1.85%/87.85%**  Human rhinovirus C **2.08%/91.90%**  SARS-CoV-2 **0.37%/99.11%** |
| W8 | 233072/7% | Rhinovirus C **1904/91.3%**  Human orthopneumovirus (Subgroup B) **5/2.1%** | Rhinovirus C **HA/40** | Human orthopneumovirus **4.28%/95.89%**  Human rhinovirus C **0.65%/99.76%**  SARS-CoV-2 **0.98%/98.97%**  Human rhinovirus 3 **0.40%/100%** |
| W7 | 207882/6% | ----------------------------------- | ---------------------------------- | Human orthopneumovirus **4.58%/95.65%** |
| W29 | 10086/5% | Human orthopneumovirus (Subgroup B) **6/2.8%** | ------------------------------ | Human orthopneumovirus **0.93%/96.48%** |
| W13 | 296436/9% | Human orthopneumovirus (Subgroup B) **382/50.9%**  Human Coronavirus NL63 **186/9.7%**  Rhinovirus B **143/26.5%** | -------------------------------------- | Coronavirus NL63 **37.88%/97.98%**  Human orthopneumovirus **63.96%/96.29%**  Human rhinovirus 3 **4.73%/95.90%**  SARS-CoV-2 **0.58%/98.66%** |
| W28 | 308324/8% | Human orthopneumovirus (Subgroup B) **30/6.3%**  Human Coronavirus NL63 **8/1.8%** | RSV **MA/57**  *Streptococcus pneumoniae* **MA/83,** Rhinovirus A **LA/4** | Human orthopneumovirus **13.48%/96.98%**  Human Coronavirus NL63 **0.70%/97.56%** |
| W11 | 245742/8% | Enterovirus B **1215/79.4%**  Coronavirus NL63 **4/0.7%**  Human orthopneumovirus (Subgroup B) **4/1.9%** | -------------------------------- | Coronavirus NL63 **0.79%/97.73%**  Human orthopneumovirus **1.81%/96.38%** |
| W36 | 178194/4% | -------------------------------- | --------------------------- | Human orthopneumovirus **1.39%/95.28%** |
| Z60 | 173128/6% | Rhinovirus A **26/4.1%**  Human respirovirus 3 **6/1.9%**  Human orthopneumovirus (Subgroup B) **4/2.2%** | *Haemophilus parainfluenzae* **MA/808** | Human orthopneumovirus **32.38%/96.72%**  Human parainfluenza virus 3 **10.53%/95.85%**  SARS-CoV-2 **0.13%/100%**  Human rhinovirus 89 **3.02%/95.2%** |
| Z1 | 230600/3% | ------------------------------------- | *Streptococcus pseudopneumoniae* **MA/2**  *Streptococcus pneumoniae* **MA/2**,  SARS-CoV-2 **LA/2** | Human orthopneumovirus **1.05%/98.12%**  SARS-CoV-2 **1.83%/98.29%** |
| Z21 | 246746/6% | Rotavirus A segment 9 **5/16.4%** | Rotavirus A **LA/7**,  *Haemophilus influenzae* **MA/15** | Human parainfluenza virus 3 **1.14%/94.47%**  SARS-CoV-2 **0.49%/99.32%** |
| A1 | 323538/8% | ------------------ | *Cryptococcus neoformans* **LA/5391** | Human rhinovirus 89 **1.02%/90.41%** |
| Z20 | 65702/2% | Human orthopneumovirus (Subgroup B) **2747/96.4%** | RSV **HA/6760** | Human orthopneumovirus **98.86%/96.79%** |
| Z19 | 189231/4% | Human orthopneumovirus (Subgroup A) **584/52.3%** | RSV **HA/721**  *Escherichia coli* **HA/546** | RSV A **87%/99%** |
| Z3 | 135972/3% | ------------------------------ | ---------------------------------- | Human orthopneumovirus **1.65%/96.88%** |
| Z5 | 182060/6% | Rhinovirus A **100/23%** | Rhinovirus A **HA/137** | Human rhinovirus 89 **5.12%/94.71%**  Human orthopneumovirus **1.89%/96.88%**  Human rhinovirus 3 **0.51%/97.30%** |
| Z6 | 138654/5% | Human orthopneumovirus (Subgroup A) **432/43%** | RSV **HA/234** | RSV A **42.9%/98.87%** |
| A37 | 269820/3% | Human orthopneumovirus (Subgroup B) **43/15.6%** | RSV **HA/71**  Rhinovirus A **LA/14** | Human orthopneumovirus **28.55%/96.57%** |
| A36 | 296462/5% | Rhinovirus A **1029/59.2%**  Enterovirus B **1262/57.8%** | Enterovirus B **HA/350**  Rhinovirus A **HA/91**. | Human rhinovirus 89 **8.49%/96.54%**  Human enterovirus 68 **1.67%/88.42%** |

LA= Low abundance, MA = Medium Abundance, HA= High Abundance, ^a^ Total reads= number of reads after removal of low quality, short or adapter containing reads. RSV= Respiratory syncytial virus;

**Supplementary Table 3:** Additional viral reads identified based on report from Genome detective.

| **Sample ID** | **HERVs** | **Phages** | **Plant viruses** | **Animal Viruses** | **Fun/Alg Viruses** | **Others** | **Sample ID** | **HERVs** | **Phages** | **Plant viruses** | **Animal viruses** | **Fun/Alg viruses** | **Others** |
| --- | --- | --- | --- | --- | --- | --- | --- | --- | --- | --- | --- | --- | --- |
| A301 |  | 232 | 25 |  | 3 |  | W43 |  | 63 |  |  |  |  |
| Z55 |  | 85 |  | 4 | 3 |  | Z35 |  | 37 |  |  |  |  |
| Z11 |  | 324 |  |  |  |  | Z45 |  | 968 |  |  |  |  |
| A29 |  | 50 |  | 2 |  |  | W16 | 720 | 7 |  |  |  |  |
| A34 |  | 250 |  |  |  |  | W34 |  | 365 |  |  |  |  |
| Z26 |  | 142 |  |  |  |  | W41 |  | 80 |  |  |  |  |
| Z58 |  | 19 |  |  | 123 |  | W40 |  | 79 |  |  |  |  |
| Z51 | 148 | 75 | 48 |  |  |  | W44 |  | 196 |  |  |  |  |
| Z38 |  | 23 |  |  |  |  | W42 |  | 347 |  |  |  |  |
| Z7 |  | 13 |  |  |  |  | W27 | 85 | 25 |  |  |  |  |
| Z34 |  | 200 |  |  |  |  | W19 |  | 248 | 2 |  |  |  |
| Z32 |  | 196 |  |  |  |  | W38 |  | 478 |  |  |  |  |
| Z49 |  | 10 |  |  | 22 |  | W32 |  | 25 |  |  |  |  |
| Z75 |  | 256 |  |  |  |  | W22 |  | 380 | 2 |  |  |  |
| Z27 | 68 | 1211 |  |  |  |  | W31 |  | 32 |  |  |  |  |
| Z44 |  | 800 |  |  |  |  | W10 |  | 373 |  |  |  |  |
| Z37 |  | 364 | 65 |  |  |  | A39 |  | 2 | 4 |  |  |  |
| Z67 |  | 23 |  |  |  |  | A28 | 89 | 11 | 6 | 2 |  |  |
| W43 |  | 89 |  |  |  |  | Z24 |  | 425 |  |  |  |  |
| Z9 |  | 421 |  |  |  |  | A25 |  | 67 | 30 |  |  |  |
| Z23 | 65 | 32 |  |  |  |  | A33 |  | 485 | 80 | 4 |  |  |
| Z15 |  | 56 |  |  |  |  | A23 |  | 615 |  |  |  |  |
| W6 |  | 37 |  |  | 242 |  | A32 |  | 2184 | 3 |  | 694 |  |
| W26 |  | 32 |  |  |  |  | A27 |  | 321 |  |  |  |  |
| W33 |  | 128 |  |  |  |  | A30 |  | 421 |  | 5 |  |  |
| W12 |  | 320 | 35 |  | 279 |  | A26 |  | 213 |  | 22393 | 7 |  |
| W3 | 12 | 253 |  | 11 |  |  | A35 | 12 | 695 |  | 55 |  |  |
| W24 |  | 65 |  | 35 |  |  | A31 |  | 102 |  |  |  |  |
| A31 |  | 200 |  | 3 |  |  | W11 |  | 32 |  |  |  |  |
| A38 |  | 110 |  | 4 | 7 |  | W36 |  | 124 |  |  |  |  |
| Z606 |  | 32 |  |  |  |  | Z60 |  | 1100 |  |  |  |  |
| A40 | 45 | 428 |  | 25 |  |  | Z1 |  | 321 |  |  |  |  |
| W1 |  | 1859 |  |  |  |  | Z21 |  | 225 |  |  |  |  |
| Z28 |  | 124 | 129 |  |  |  | A1 |  | 205 |  |  |  |  |
| Z53 |  | 1596 |  |  | 35 |  | Z20 | 22 | 16 |  |  |  |  |
| Z76 |  | 369 |  |  |  |  | Z19 |  | 88 |  |  |  |  |
| Z78 |  | 258 |  |  |  |  | Z3 |  | 222 |  |  |  |  |
| Z16 |  | 632 |  |  |  |  | Z5 |  | 16 |  |  |  |  |
| W14 |  | 120 |  |  |  |  | Z6 |  | 78 |  |  |  |  |
| W8 |  | 257 |  |  |  |  | A37 |  | 300 |  |  | 185 |  |
| W7 | 12 | 97 |  |  |  |  | A36 |  | 143 |  | 6 |  |  |
| W29 |  | 6 |  |  |  |  | W28 | 23 | 64 |  |  | 65 |  |
| W13 |  | 175 |  | 33 |  | 2 |  |  |  |  |  |  |  |

Fun=Fungal; Alg= Algae; Phages= Bacteriophages.

**HERV**: Human endogenous retroviruses K113.

**Bacteriophages**: Sum of phages from *Proteus, Enterobacteria, Escherichia, Propionibacterium, Sulfitobacter, Klebsiella, Lambdavirus lambda,  Lactococcus, Shigella, Vibrio, Klebsiella*.

**Plant Viruses:** Sum of viruses from Maize associated totivirus, Bermuda grass latent virus, Apple chlorotic leaf spot virus, Apricot pseudo-chlorotic leaf spot virus, Phytophthora parasitica virus, Tobacco mosaic virus, Carrot cryptic virus, Fig cryptic virus, Red clover powdery mildew associated totivirus,

**Animal viruses:** Sum of viruses from Chicken picobirnavirus (segment RNA 2), Equine infectious anemia virus, Avian carcinoma virus, Canarypox virus, Saimiriine gamma herpesvirus 2, Feline leukemia virus,

**Fun/Alg viruses**: Sum of viruses from Scheffersomyces segobiensis virus L, Diatom colony associated virus,

**Others:** Circovirus-like genome DCCV-4

**Supplementary Table 4.** Detected viral and bacterial reads in the negative control across the three mNGS analysis tools

| Contaminants in NFW | One Codex | Genome Detective | Twist Panel |
| --- | --- | --- | --- |
| Viral reads | -------------------------- | ------------------------- | ------------------------- |
| Bacteria reads at medium abundance | *Streptococcus oralis, Veillonella sp. oral taxon 780, Kocuria rosea, Anaerococcus senegalensis, Cloacibacterium* normanense, Anaerococcus prevotii, *Streptococcus sp.* 263_SSPC, *Clostridium tepidum*  4 | N/A | N/A |
| Bacteria reads at low abundance | *Escherichia coli*  *Staphylococcus epidermidis*  *Staphylococcus aureus*  *Acinetobacter baumannii*  *Streptococcus mitis* | N/A | N/A |

## Supplementary Table 5: A summary of the number of influenza cases detected per province in South Africa during the study period (Week 50 of 2020 - Week 34 of 2021) as reported in the NICD weekly respiratory pathogens surveillance report. (<https://www.nicd.ac.za/diseases-a-z-index/disease-index-covid-19/surveillance-reports/weekly-respiratory-pathogens-surveillance-report-week/>)

|  | Week 50 2020 | Week 1-14 2021 | Week 15-16 2021 | Week 17-19 2021 | Week 20-34 2021 |
| --- | --- | --- | --- | --- | --- |
| EC | 0 | 0 | 0 | 0 | 1 |
| WC | 1 | 0 | 0 | 0 | 8 |
| GAUTENG | 0 | 1 | 0 | 3 | 23 |
| FS | 0 | 0 | 0 | 0 | 0 |
| LIMPOPO | 0 | 0 | 0 | 0 | 0 |
| MP | 0 | 0 | 0 | 0 | 0 |
| NW | 0 | 0 | 0 | 1 | 8 |
| NC | 0 | 0 | 0 | 0 | 0 |
| KZN | 0 | 0 | 6 | 0 | 8 |

EC=Eastern Cape; WC= Western Cape; FS= Free State; MP= Mpumalanga; NW= North West; NC= Northern Cape; KZN= KwaZulu Natal.
